# Lack of FcRn Impairs Natural Killer Cell Development and Functions in the Tumor Microenvironment

**DOI:** 10.3389/fimmu.2018.02259

**Published:** 2018-09-28

**Authors:** Diana Cadena Castaneda, Christine Dhommée, Thomas Baranek, Emilie Dalloneau, Laurie Lajoie, Alexandre Valayer, Christophe Arnoult, Marie-Véronique Demattéi, Delphine Fouquenet, Christelle Parent, Nathalie Heuzé-Vourc'h, Valérie Gouilleux-Gruart

**Affiliations:** ^1^Université François Rabelais de Tours, Tours, France; ^2^CNRS, GICC UMR 7292, Tours, France; ^3^INSERM, Centre d'Etude des Pathologies Respiratoires, U1100, Tours, France

**Keywords:** FcRn, anti-tumor immunity, NK cells, IFN-γ, CD107a, *fcgrt* knock-out

## Abstract

The neonatal Fc receptor (FcRn) is responsible for the recycling and transcytosis of IgG and albumin. FcRn level was found altered in cancer tissues and implicated in tumor immunosurveillance and neoplastic cell growth. However, the consequences of FcRn down-regulation in the anti-tumor immune response are not fully elucidated. By using the B16F10 experimental lung metastasis model in an FcRn-deficient microenvironment (FcRn^−/−^ mice), we found lung metastasis associated with an abnormal natural killer (NK) cell phenotype. In FcRn^−/−^ mice, NK cells were immature, as shown by their surface marker profile and their decreased ability to degranulate and synthesize interferon γ after chemical and IL-2 or IL-12, IL-15 and IL-18 activation. These new findings support the critical role of FcRn downregulation in the tumor microenvironment in anti-tumor immunity, via NK cell maturation and activation.

## Introduction

The neonatal Fc receptor (FcRn) is a member of the IgG-Fc receptor family comprising type I (e.g., “classical” FcγRs) and type II (e.g., non-classical FcR: FcRn, TRIM21) receptors (1–3). The structure, expression and functions of this IgG-Fc receptors have been extensively rewiewed regarding their major role in the regulation of immune responses (4). FcRn is an MHC class I-related molecule consisting of a heavy chain associated with β2-microglobulin molecule, well-known for its role in regulating IgG and albumin homeostasis (5). Indeed, FcRn-dependent IgG and albumin recycling leads to an extended half -life of these two molecules (6, 7). FcRn is also a main actor in the biodistribution of IgG and albumin throughout the body, via transcytosis (3, 8). Accordingly, FcRn expression is ubiquitous within organs and tissues, with high expression in endothelial and epithelial cells (9). It is also expressed by hematopoietic cells, in particular macrophages/monocytes and dendritic cells (DCs) (10). The expression of FcRn in antigen-presenting cells is connected to its implication in the humoral immune response, via an immune complex presentation (11).

Besides these functions, FcRn was recently found an important player in anti-tumor immunity. First, FcRn in immune cells was shown to be critical for the activation of tumor-reactive CD8^+^ T cells in colorectal cancer (12). The density of FcRn-expressed DCs was correlated with CD8^+^ T-cell number and predicted improved prognosis in human colorectal carcinoma. Second, we reported FcRn mRNA and protein levels in both lung cancerous tissue and non-cancerous tissue associated with favorable prognosis in non-small cell lung cancer (13). Third, studies involving neoplastic cells expressing different levels of FcRn showed that FcRn-mediated recycling of albumin reduced tumor cell growth and proliferation (14).

Because FcRn may shape additional anti-tumor properties, here we further investigated the consequences of its downregulation. We used the B16F10 experimental lung metastasis model (15, 16) in an FcRn-depleted environment (FcRn^−/−^ mice) and compared the different cellular components of the anti-tumor immune response in wild-type (WT) and FcRn^−/−^ mice. We explored natural killer (NK) cells as relaying FcRn-dependent anti-tumor immunity. These cells are important in the host and therapy-induced immune response against cancer (17, 18) and their efficacy *in vivo* is compromised by suppressive signals delivered by tumor or tumor-associated cells (19, 20).

## Materials and methods

### Cell line

The murine melanoma cell line B16F10 Luc^+^ was a kind gift from Dr Laurent Gros (Institute of Cancer Research of Montpellier, Montpellier, France). The murine lymphoma cell line YAC-1 was purchased from the American Type Culture Collection (ATCC). B16F10 Luc^+^ and YAC-1 cells were maintained in RPMI 1640 culture medium (Sigma-Aldrich) supplemented with 10% heat-inactivated FBS (Lonza), 2 mM L-glutamine, 100 U/ml penicillin and 100 μg/ml streptomycin (Sigma-Aldrich) referred as complete medium.

### B16F10 experimental lung metastasis model

WT C57BL/6J mice were obtained from Charles River Laboratories. FcRn^−/−^ C57BL/6J mice, deficient in *fcgrt* gene (B6.129X1-Fcgrt tm1 Dcr/DcrJ (fcgrt^−/−^)], were originally purchased from The Jackson Laboratory. A targeting vector was designed to replace 1,588 nucleotide fragments (encoding the promoter sequence 5′ end of the transcriptional start site, exon 1, intron 2, and most of exon 2) with a PGK-NeoR cassette. The vector was electroporated into 129X1/SvJ-derived ESV/J-1182 embryonic stem (ES) cells. Correctly targeted ES cells were injected into recipient C57BL/6J blastocysts. The resulting chimeric animals were crossed to C57BL/6J mice. The mice were then backcrossed to C57BL/6J for 11 generations. All mice were maintained in a dedicated pathogen-free environment in our animal facility and were used at age 7–12 weeks. All animal studies were performed according to French national regulations (EC directive 86/609/CEE, French decree no. 87-848) after approval was received from the Committee on the Ethics of Animal Experiments of the Val-de-Loire, CEEA VdL (referral no. 2015070117414040).

Syngeneic experimental lung metastases were induced by intravenously injecting 10^5^ B16F10 Luc^+^ melanoma cells in 100 μl RPMI 1640 medium in the tail vein of WT and FcRn^−/−^ mice. The cells colonized lungs and formed well-defined black melanocytic nodules in the lung (15, 21). After 18 days, mice were euthanized. Lungs and spleens were harvested for further analysis. Lungs were scored for number of tumor nodules.

### Cell preparation for flow cytometry

Lungs were dissociated into single-cell suspensions by combining mechanical dissociation (gentleMACS Dissociators, Miltenyi) with enzymatic degradation of the extracellular matrix. The enzymatic degradation involved use of a digestion buffer: RPMI 1640 containing 5% FBS, 125 μg/ml Liberase LT (Roche Diagnostics) and 100 μg/ml DNAse corresponding to 200 Kuntz units/ml DNAse (Roche Diagnostics). Spleens were flushed with a 25G needle and syringe containing the digestion buffer, then incubated for 30 min at 37°C. Bone-marrow cells were isolated from the femur and tibia by flushing with a 25G needle and syringe containing 1X PBS. Red blood cells in the resulting cell suspensions were lysed by using an ammonium chloride buffer, then filtered (70 μm, MACS SmartStrainers) and resuspended at 10^7^ cells/ml in 1X PBS containing 5% FBS and 2 mM EDTA.

### Murine NK-cell isolation and *ex vivo* expansion and stimulation

NK cells were isolated from pooled spleens of WT or FcRn^−/−^ mice by negative selection with magnetic Microbeads (NK cell isolation kit II, Miltenyi). NK-cell purity was >90%. NK cells were expanded in RPMI 1640 complete medium supplemented with 5,000 U/ml rhIL2 (PreproTech) for 4 days. For the cell growth and mortality analyses, cells were counted daily using a cell counting chamber (Malassez). Cell surface-mobilized CD107a and intracellular IFN-γ levels were measured as described (22) after 4-h stimulation with phorbol 12-myristate 13-acetate (PMA, 100 ng/mL) and 500 ng/mL ionomycin calcium (Sigma-Aldrich) at 37°C in 5% CO_2_. Next, 10^5^ freshly isolated NK cells were added per well with 5 μl (0.5 mg/ml) anti-CD107a (clone 1D4B, FITC, Becton Dickinson [BD]) and 1 μg/10^6^ cells of brefeldin A (GolgiPlug, BD). At the end of incubation, cells were washed with 1X PBS containing 5% FBS and 2 mM EDTA. Then, NK cells were treated with an Fc block (anti-CD32/CD16 in the form of 2.4G2 hybridoma culture supernatant) to inhibit non-specific antibody binding for 20 min and stained for surface NK-cell markers: NK1,1 (clone PK136, APC, BD), NKp46 (clone 29A1.4, Pe, BD), CD27 (clone LG.3A10, PeCy7, BD) and CD11b (clone M1/70, PerCPCys5.5, BD) for 30 min. Samples were fixed and permeabilized according to the manufacturer's instructions (BD) and stained for intracellular IFN-γ (clone XMG1.2, APC-H7, BD) for an additional 30 min. After washing, cells were analyzed by flow cytometry. For cytokine induced cell surface-mobilized CD107a and intracellular IFN-γ production, 2 × 10^6^–5 × 10^6^ freshly isolated splenocytes were seeded in RMPI 1640 complete medium supplemented or not with 5,000 U/ml rhIL2 or 5 ng/ml rhIL12 (MBL), 50 ng/ml rhIL15 (Miltenyi) and 10 ng/ml rhIL18 (MBL) for 4-h in the presence of brefeldin A and anti-CD107a. After incubation, the cells were additionally stained for CD3, NKp46, NK1.1 and IFN-γ flow cytometry analysis.

### Murine NK-cell cytotoxicity assay

For *in vitro* cytotoxicity assays, isolated NK cells were maintained overnight in RPMI 1640 complete medium supplemented with 50 U/ml rhIL2 and mixed at indicated effector: target ratios (10:1, 5:1 and 1:1) with carboxyfluorescein diacetate succinimidyl ester (CFSE, TonboBio) labeled target YAC-1 cells as previously described (23, 24). After 4-h of incubation at 37°C, the specific target cell lysis was assessed by flow cytometry (23).

### Flow cytometry

Single-cell suspension prepared from mouse lungs and spleens were analyzed by flow cytometry (MACSQuant Analyzer 10, Miltenyi). After adding Fc blocking 2.4G2 hybridoma supernatant, cells were stained with mixtures of monoclonal antibodies at the optimal concentration determined in our laboratory and designed to distinguish leukocytes. Mix 1, for dendritic cells, neutrophils and macrophage/monocytes: CD45 (clone 30-F11, APC-H7, BD), CD11c (clone N418 APC, BD), CD11b (PErCPCys5.5, BD), Ly6G (clone 1A8, FITC, eBioscience [eBio]), Ly6C (clone HK1.4, PeCy7, eBio), and F4/80 (clone BM8, V450, BD). Mix 2, for B and T lymphocytes: CD45, CD19 and B220 (clone RA3-6B2, APC, BD), CD3 and TCR (clone H57-597, V450, BD), CD8 (clone 53-6.7, PerCPCys5.5, BD) and CD4 (clone GK1.5, PeCy7, eBio). Mix 3, for NK cells: CD45, exclusion of T and B cells (lineage CD3/TCR/CD19/B220, V450), NKp46 (PE, eBio); and for NK maturation, CD27 (APC, BD) and CD11b (PErCPCys5.5, BD). For bone-marrow single-cell suspensions, an exclusive additional mix was used for NK development: CD45, exclusion of T and B cells (lineage CD3/TER119/C19/B220, FITC), CD122 (clone TM-beta 1, Pe, eBio), NK1,1 (PerCPCys5.5, BD), NKp46 (V450, BD), CD11b (PeCy7, BD), and CD49b (clone DX5, APC, BD). LIVE/DEAD Fixable Aqua Dead Cell Stain Kit (Invitrogen) was added for excluding nonviable cells in all analyses. Data analysis involved use of Kaluza 1.3 flow cytometry analysis software (Beckman Coulter).

### Statistical analysis

All results are expressed as median ± Min to Max, unless specified. Statistical significance was analyzed by two-tailed non-parametric and unpaired Wilcoxon-Mann-Whitney test. *P* < 0.05 was considered statistically significant. Statistical analysis involved use of Prism 5 (GraphPad Software, San Diego, CA), where, ns = not significant, ^*^p ≤ 0.05, ^**^p ≤ 0.005 and ^***^p ≤ 0.0001.

## Results

### Abnormal immune cell profile associated with development of experimental lung metastasis in mice lacking FcRn

**Figure 1:**
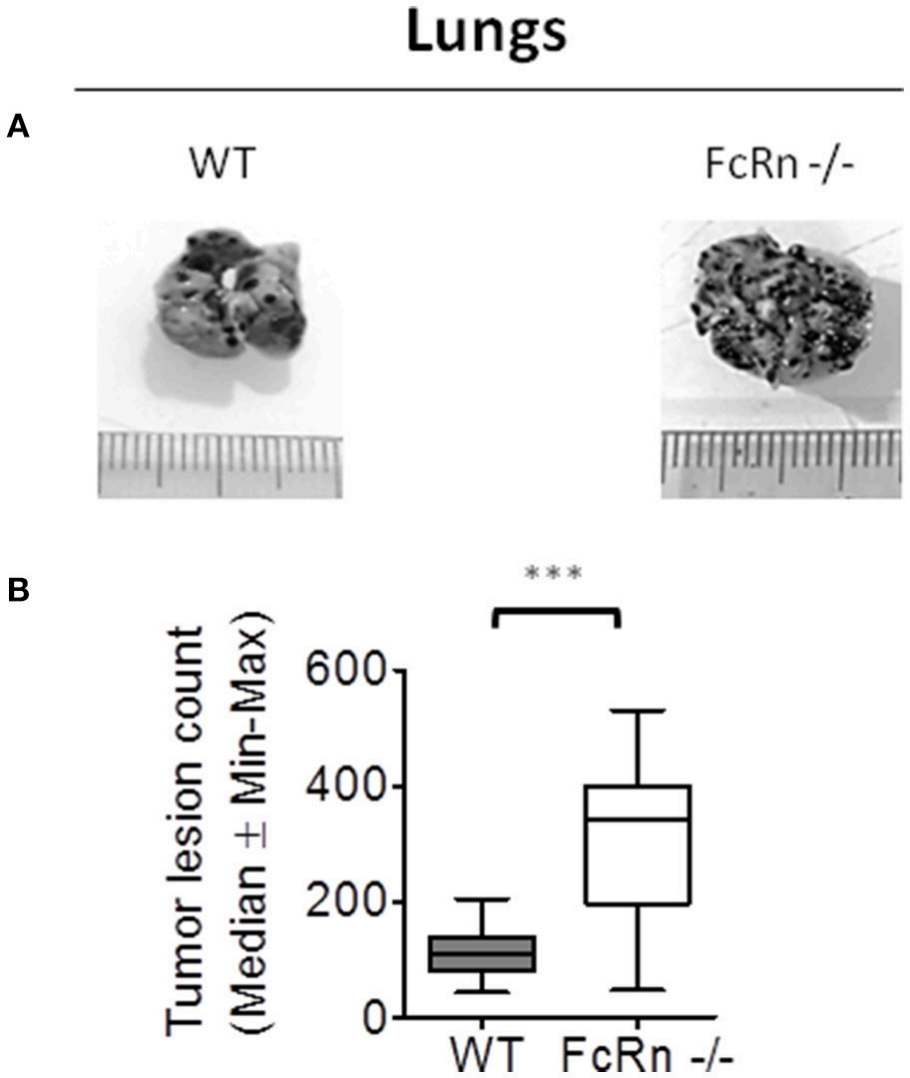
Role of neonatal Fc receptor (FcRn) in experimental lung metastasis development. Wild-type (WT) (*n* = 14) and FcRn^−/−^ (*n* = 12) mice were intravenously injected in the tail vein with 1 × 10^5^ B16-F10 tumor cells in 100 μl medium. 18 days after tumor injection, animals were sacrificed and lung tumor lesions were blindly counted. **(A)** Representative photographs of WT and FcRn^−/−^ lungs after 18 days. **(B)** Nodule counts in WT and FcRn^−/−^ mice. Data are expressed as median ± Min to Max from three independent experiments. ^***^p ≤ 0.0001 using two-tailed non-parametric and unpaired Wilcoxon-Mann-Whitney test.

To dissect the role of FcRn in anti-tumor immunity, we used the well-characterized syngeneic B16F10 experimental lung metastasis mouse model (15, 16). B16F10 cells were intravenously injected in WT and FcRn^−/−^ C57BL/6 mice. Macroscopy of lungs revealed a greater number of pulmonary nodules in FcRn^−/−^ than WT mice at 18 days post-implantation (Figure 1). No nodule was detected in other organs under the experimental conditions.

**Figure 2:**
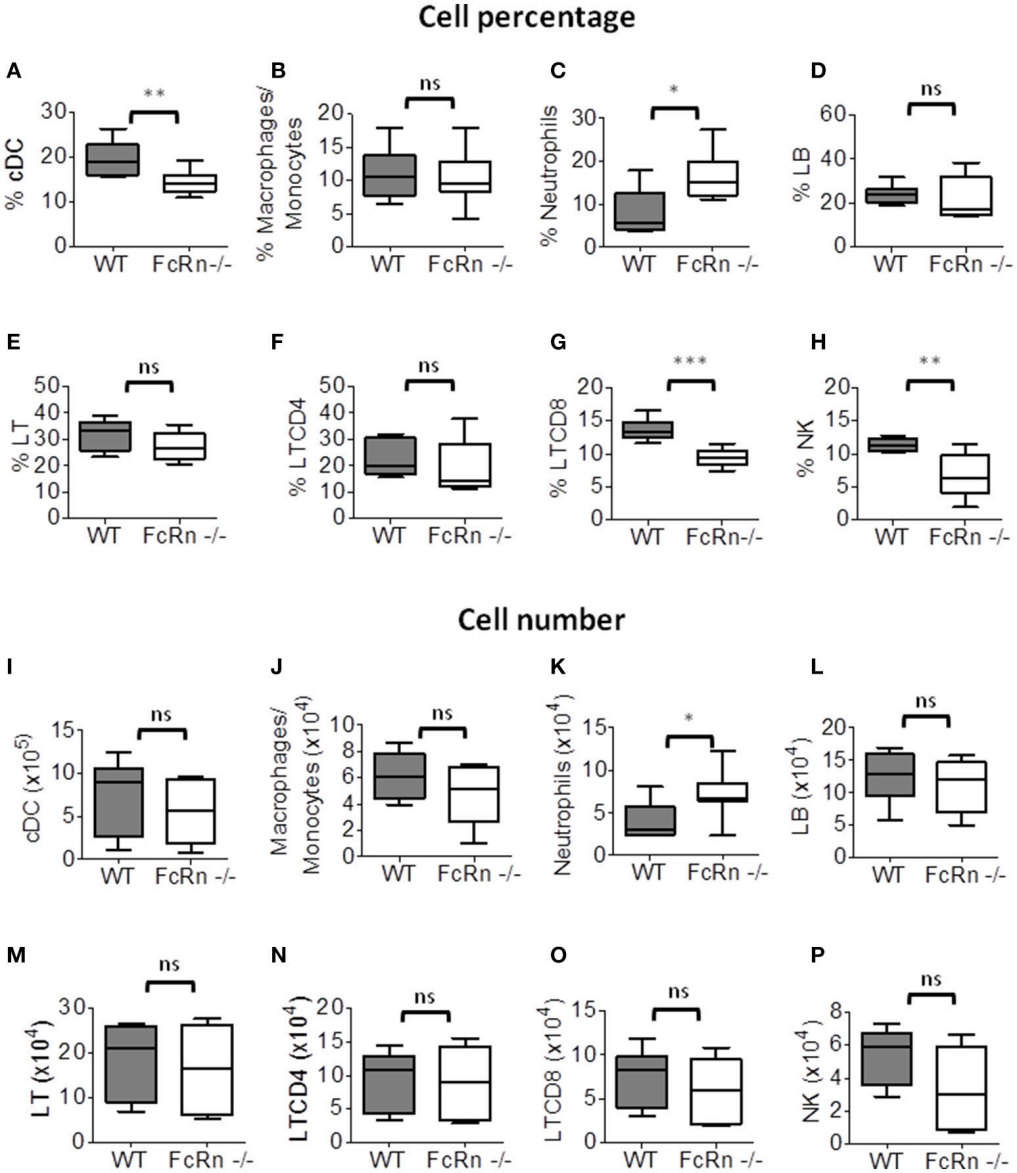
Flow cytometry of leukocytes in lungs of WT (*n* = 8) and FcRn^−/−^ (*n* = 8) mice injected, in the tail vein, with 1 × 10^5^ B16-F10 tumor cells in 100 μl medium. Lungs were excised and dissociated by combining mechanical dissociation with enzymatic degradation of the extracellular matrix to obtain a single-cell suspension and cells were resuspended at 10^7^ cells/ml in 1X PBS containing 5% FBS and 2 mM EDTA for flow cytometry staining. Results correspond to the proportion (top panel: **A–H**) and number (bottom panel: **I–P**) of **(A,I)** conventional dendritic cells, **(B,J)** macrophages/monocytes, **(C,K)** neutrophils, **(D,L)** B lymphocytes, **(E,M)** T lymphocytes, **(F,N)** CD4 T lymphocytes, **(G,O)** CD8 T lymphocytes, and **(H,P)** natural killer (NK) cells (See Figure S1 for gating strategy). Data are expressed as median ± Min to Max from one out of three independent experiments with similar results. ns = not significant, ^*^p ≤ 0.05, ^**^p ≤ 0.005, and ^***^p ≤ 0.0001 using two-tailed non-parametric and unpaired Wilcoxon-Mann-Whitney test.

To investigate the immune cell populations in lungs of WT and FcRn^−/−^ mice, we used flow cytometry of cell suspensions from fully dissociated lungs to distinguish leukocyte populations (Supplementary Figure S1). The proportion of macrophages/monocytes (CD11b^+^/F4/80^+^), B lymphocytes (CD19^+^/B220^+^), T lymphocytes (CD3^+^/TCR^+^) and CD4^+^ T lymphocytes (CD3^+^/TCR^+^/CD4^+^) did not differ between FcRn^−/−^ and WT mice (Figures 2B–F), but that of conventional dendritic cells (cDCs: CD11c^+^/CD11b^+^) and CD8^+^ T lymphocytes (CD3^+^/TCR^+^/CD8^+^) was significantly lower in FcRn^−/−^ than WT mice (Figures 2A,G). As well, the percentage of neutrophils (CD11b^+^/Ly6G^+^) was significantly higher in FcRn^−/−^ than WT mice (Figure 2C). The proportion of NK cells (CD3^−^/B220^−^/NKp46^+^), which do not express FcRn (Supplementary Figure S2), was significantly decreased in FcRn^−/−^ mice (Figure 2H). As well, the number of NK cells was altered but not significantly (*p* = 0.059) in FcRn^−/−^ mice (Figure 2P). The amount of other cell types was not affected (Figures 2I,J,L–O), except for neutrophils, which were increased in FcRn^−/−^ mice (Figure 2K).

**Figure 3:**
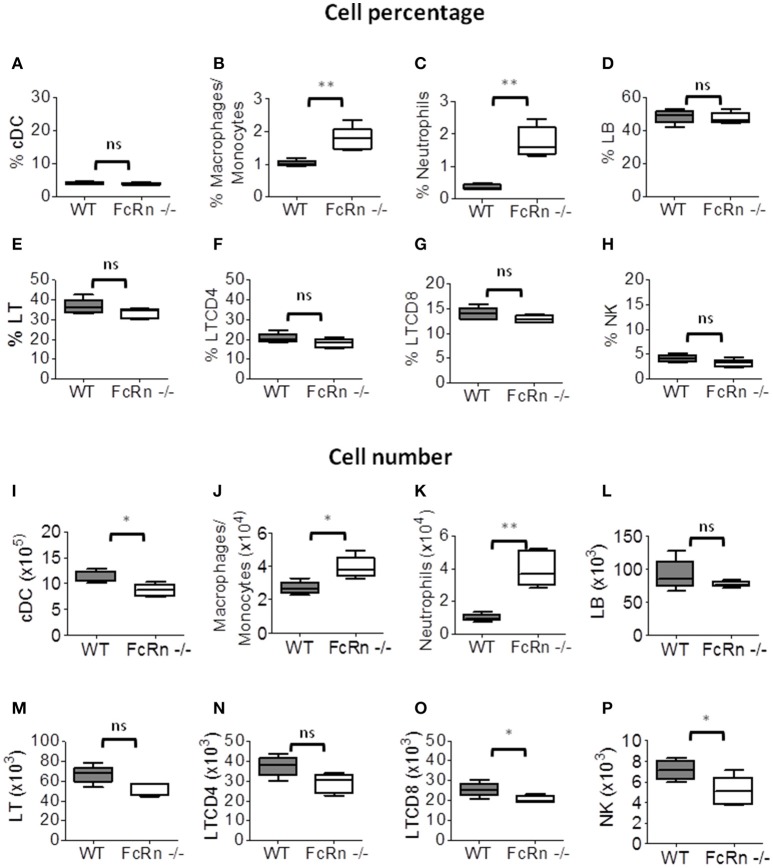
Flow cytometry of leukocytes in the spleen of WT (*n* = 5) and FcRn^−/−^ (*n* = 5) mice injected, in the tail vein, with 1 × 10^5^ B16F10 tumor cells in 100 μl medium. Spleens were recovered and dissociated by combining mechanical dissociation with enzymatic degradation of the extracellular matrix to obtain a single-cell suspension. Then, cells were resuspended at 10^7^ cells/ml in 1X PBS containing 5% FBS and 2 mM EDTA for flow cytometry staining. Results correspond to the proportion (top panel: **A–H**) and number (bottom panel: **I–P**) of **(A,I)** conventional dendritic cells, **(B,J)** macrophages/monocytes, **(C,K)** neutrophils, **(D,L)** B lymphocytes, **(E,M)** T lymphocytes, **(F,N)** CD4 T lymphocytes, **(G,O)** CD8 T lymphocytes, and **(H,P)** NK cells. Data are expressed as median ± Min to Max from one of two independent experiments with similar results. ns = not significant, ^*^p ≤ 0.05 and ^**^p ≤ 0.005 using two-tailed non-parametric and unpaired Wilcoxon-Mann-Whitney test.

Because the lungs are the main primary site of B16F10 nodules after intravenous injection, we explored whether the cell modifications were also detected in other peripheral organs, such as the spleen, where no nodule was detected under our experimental conditions. In the spleen, the percentage and number of macrophages/monocytes (Figures 3B,J) and neutrophils (Figures 3C,K) were significantly greater in FcRn^−/−^ than WT mice. There was no difference in percentage and number of B lymphocytes (Figures 3D,L), T lymphocytes (Figures 3E,M) and CD4^+^ T lymphocytes (Figures 3F,N). The numbers of cDCs, CD8^+^ T lymphocytes and NK cells were decreased in FcRn^−/−^ mice (Figures 3I,O,P), with no variation in proportion of these cells (Figures 3A,G,H). Altogether, data obtained in this experimental lung metastasis model are consistent with the implication of cDCs, CD8^+^ T lymphocytes and NK cells in tumor development in FcRn^−/−^ mice.

### Lack of FcRn affects NK cell maturation in experimental lung metastasis model

**Figure 4:**
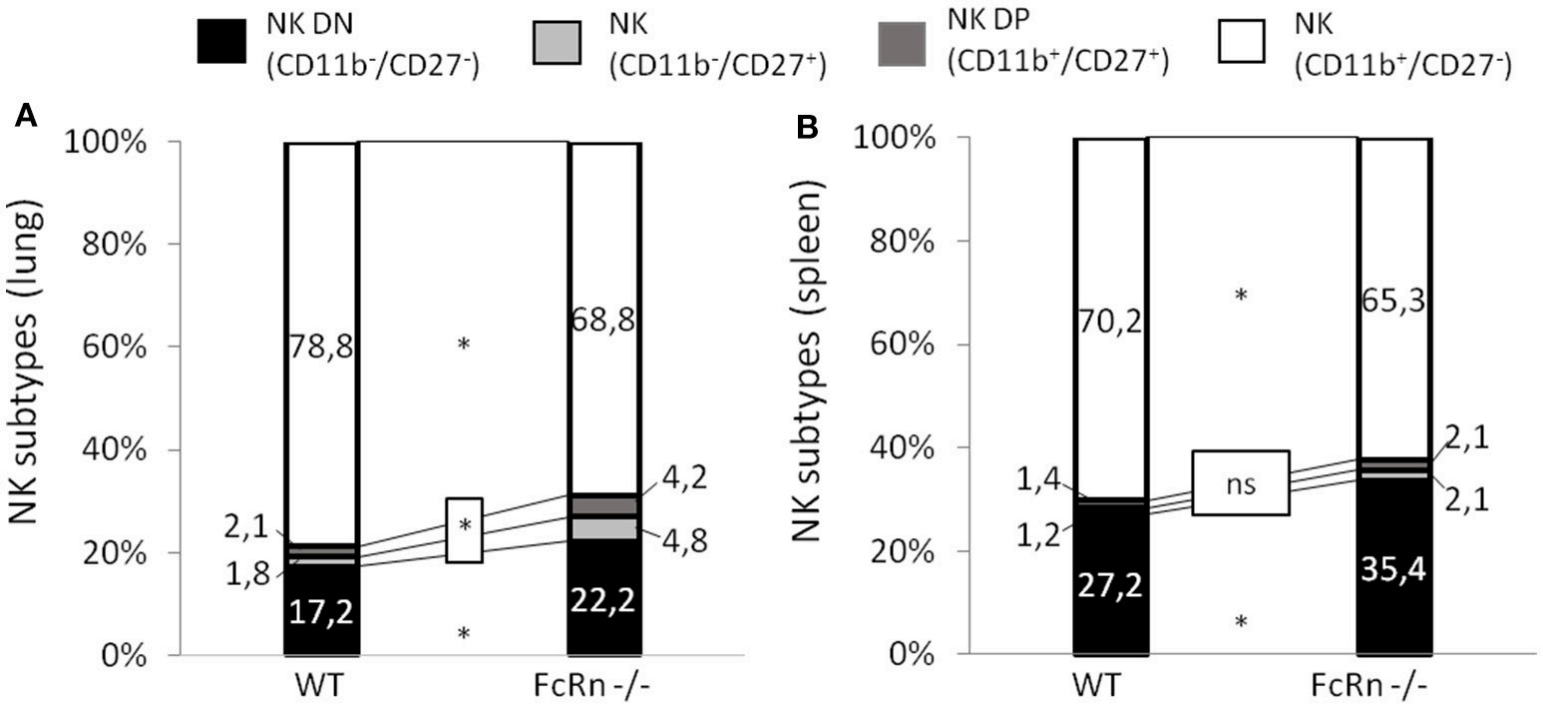
Flow cytometry of NK subtypes based on CD11b and CD27 markers in **(A)** lungs and **(B)** spleen from WT (*n* = 10) and FcRn^−/−^ (*n* = 10) mice injected, in the tail vein, with 1 × 10^5^ B16F10 cells in 100 μl medium. Lungs and spleen were collected from euthanized animals and dissociated by combining mechanical dissociation with enzymatic degradation of the extracellular matrix to obtain a single-cell suspension. Then, cells were resuspended at 10^7^ cells/ml in 1X PBS containing 5% FBS and 2 mM EDTA for flow cytometry staining. Histograms represent the sum of the percentage of CD11b^−^/CD27^−^, CD11b^−^/CD27^+^ (CD11b^−^), CD11b^+^/CD27^+^, and CD11b^+^/CD27^−^ NK cell subtypes in WT and FcRn^−/−^ mice. Data are means from two independent experiments. ns = not significant, ^*^p ≤ 0.05 using two-tailed non-parametric and unpaired Wilcoxon-Mann-Whitney test.

To gain insight into defective NK cells in an FcRn-depleted microenvironment, we explored NK cell maturation by flow cytometry on cell suspensions from lungs and spleen of mice implanted with B16 cells. We distinguished the different stages of NK-cell maturation as previously described under physiological conditions (25, 26). In peripheral organs, mouse NK-cell maturation proceeds through four stages based on CD11b/CD27 expression: CD11b^−^/CD27^−^ (double negative: DN), CD11b^−^/CD27^+^ (CD11b^−^), CD11b^+^/CD27^+^ (double positive: DP), and CD11b^+^/CD27^−^ (CD27^−^). We analyzed these different NK subtypes in lungs and spleen of mice (Supplementary Figure S3). In lungs, the proportion of DN, CD11b^−^ and DP NK cells was significantly greater in FcRn^−/−^ than WT mice, whereas that of CD27^−^ NK cells, the more mature NK stage, was significantly decreased (Figure 4A). The sum of DN, CD11b^−^, and DP NK cells, corresponding to the less mature cells, was 31.2 and 21.1% in FcRn^−/−^ and WT mice, respectively. Similarly, in spleen, the proportion of less mature cells DN NK cells but not CD11b^−^ and DP NK cells was greater in FcRn^−/−^ than WT mice. The proportion of CD27^−^ NK cells was lower in FcRn^−/−^ than WT mice (Figure 4B). These results reveal less mature NK cells in FcRn-deficient than WT mice in the B16F10 lung metastasis model, with a more pronounced effect in the lungs.

### NK cell development/maturation is impaired in FcRn^−/−^ naive mice

**Figure 5:**
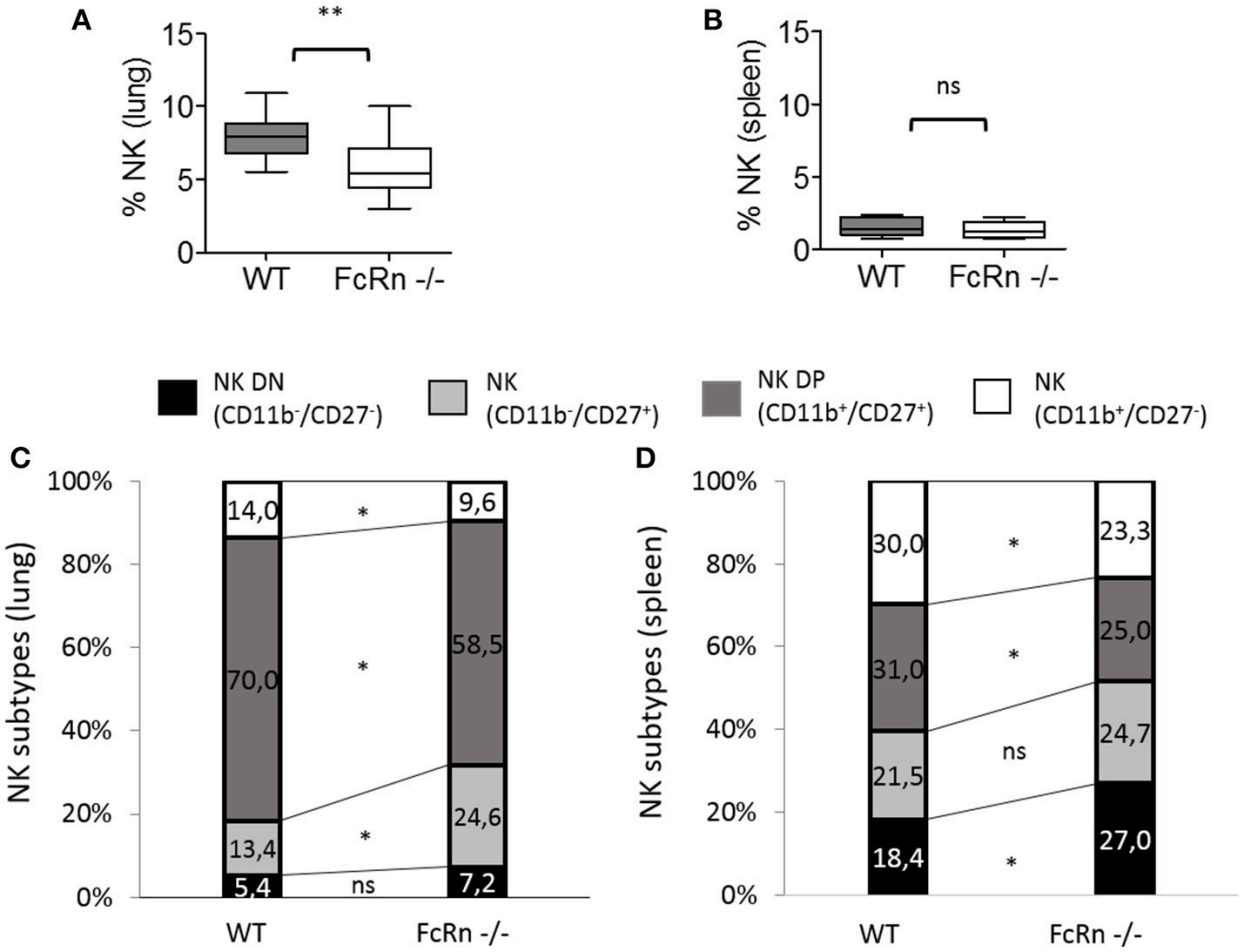
Flow cytometry of NK cell proportion in **(A)** lungs and **(B)** spleen from WT (*n* = 13) and FcRn^−/−^ (*n* = 12) naive mice. Data are expressed as median ± Min to Max from two independent experiments. Flow cytometry of NK subtypes based on CD11b and CD27 markers in **(C)** lungs and **(D)** spleen from WT (*n* = 5) and FcRn^−/−^ (*n* = 5) naive mice. After euthanasia of naive animals, lungs and spleen were collected and dissociated by combining mechanical dissociation with enzymatic degradation of the extracellular matrix to obtain a single-cell suspension. Then, cells were resuspended at 10^7^ cells/ml in 1X PBS containing 5% FBS and 2 mM EDTA for flow cytometry staining. Histograms represent the sum of the percentage of CD11b^−^/CD27^−^, CD11b^−^/CD27^+^ (CD11b^−^), CD11b^+^/CD27^+^, and CD11b^+^/CD27^−^ NK cell subtypes in WT and FcRn^−/−^ mice. Data are means from one experiment. ns = not significant, ^*^p ≤ 0.05, ^**^p ≤ 0.005 using two-tailed non-parametric and unpaired Wilcoxon-Mann-Whitney test.

To establish whether the defective NK cell maturation was consecutive to B16F10 cell injection or pre-existed in FcRn^−/−^ mice, we phenotyped leukocytes by flow cytometry in naive mice (Supplementary Figures S4, S5) and focused on NK-cell sub-populations. First, the proportion of NK cells in lungs was significantly lower in FcRn^−/−^ than WT mice (Figure 5A), with no difference in spleen (Figure 5B). Second, phenotype analysis of NK cells revealed an organ-specific NK subtype distribution previously described (27, 28) corresponding to an increased NK DP population in lungs vs. spleen in both WT and FcRn^−/−^ mice (Figures 5C,D). Third, the proportion of DN and CD11b^−^ NK cells, corresponding to more immature cells, was greater in lungs and spleen of FcRn^−/−^ than WT mice (31.8 vs. 18.8% in lungs and 51.7 vs. 39.9% spleen). Altogether, these results suggest that defective NK cell development/maturation was already present in naive FcRn^−/−^ mice.

**Figure 6:**
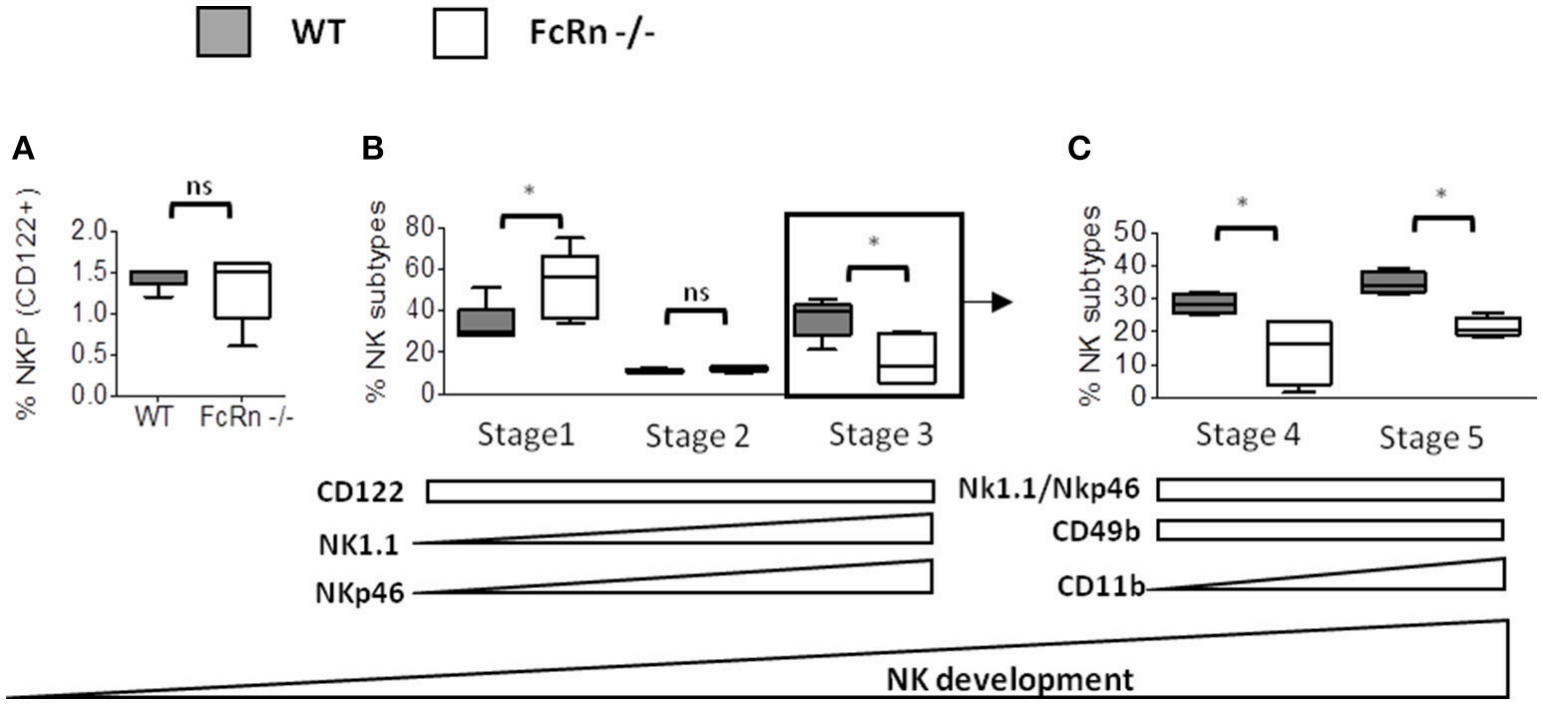
Flow cytometry of NK cell development in bone marrow of WT (*n* = 5) and FcRn^−/−^ (*n* = 5) naive mice. After euthanasia of naive animals, bone-marrow cells were isolated from the femur and tibia and cells separated by mechanical dissociation. Then, cells were resuspended at 10^7^ cells/ml in 1X PBS containing 5% FBS and 2 mM EDTA for flow cytometry staining. The development of NK cells is initiated with the expression of **(A)** CD122, then three early immature steps [NK precursor (NKP), stage 1 and stage 2] are identified with the expression of **(B)** CD122, NK1,1 and NKp46. **(C)** From stage 2, stage 5 and 6 subtypes are characterized by CD49b and CD11b expression. Acquisition of marker during NK cell development is represented schematically by arrows under the graphics. Data are expressed as median ± Min to Max from one out of two independent experiments with similar results. ns = not significant and ^*^p ≤ 0.05 using two-tailed non-parametric and unpaired Wilcoxon-Mann-Whitney test.

To further investigate the origin of the impaired NK cell maturation in an FcRn-depleted environment, we compared NK-cell development in bone marrow of FcRn^−/−^ and WT mice. To distinguish the different stages of NK cell development in bone marrow, we used specific markers as previously described (29). The global level of NK cell precursors in bone marrow did not differ between FcRn^−/−^ and WT mice, as characterized by CD122 expression, also known as interleukin-2Rβ (Figure 6A). In contrast, the following stages of NK-cell development were altered. The proportion of NK cells in stage 1 (CD122^+^/NK1.1^−^/NKp46^−^) was increased in FcRn^−/−^ mice and that of NK cells in stages 3, 4, and 5 was decreased as compared with WT mice (Figures 6B,C). Our results clearly indicate significantly impaired NK cell development in FcRn^−/−^ mice.

### Defective functions of NK cells from FcRn^−/−^ mice

**Figure 7:**
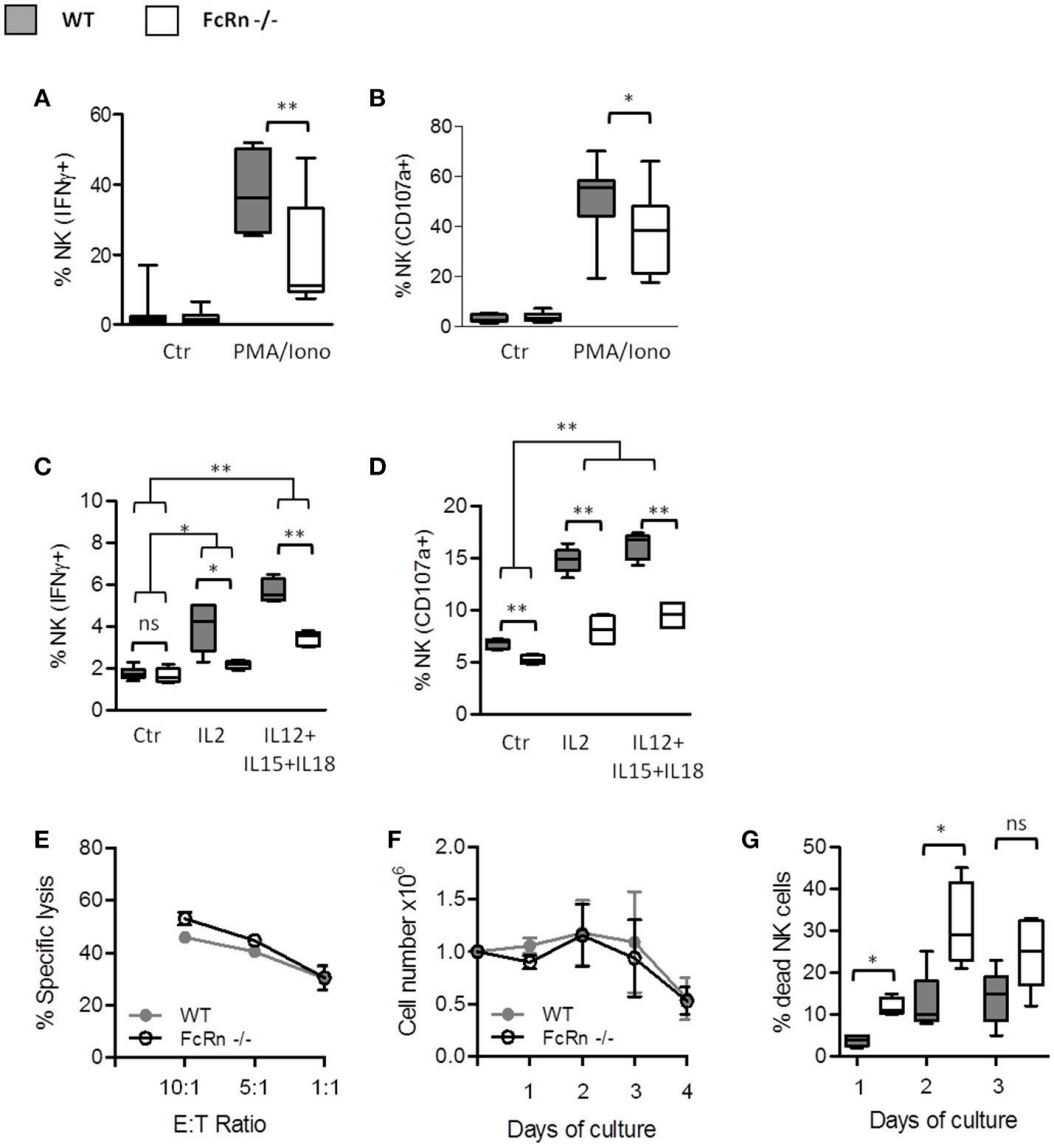
Influence of FcRn on NK cell functions and *in vitro* expansion. Purified splenic NK cells were analyzed by flow cytometry for **(A)** the intracellular measurement of IFN-γ and **(B)** the surface expression of the late endosomal marker CD107a, after 4-h incubation at 37°C without (Ctr) or with PMA (100 ng/mL)/ionomycine (500 ng/mL). Data are median ± Min to Max analyzed from eight independent experiments using pooled NK cells from 2 mice. Freshly isolated splenocytes were seeded in RMPI 1640 complete medium supplemented with 5,000 U/ml rhIL2 or with 5 ng/ml rhIL12, 50 ng/ml rhIL15, and 10 ng/ml rhIL18 for 4-h **(C,D)**. Within splenocytes, CD3^−^/NK1.1^+^/NKp46^+^ cells were analyzed for **(C)** the intracellular expression of IFN-γ and **(D)** the surface expression of CD107a by flow cytometry. Data are median ± Min to Max from two independent experiments using pooled spleens from 2 mice. **(E)** Cytotoxicity assay was performed against CFSE-labeled YAC-1 target cells with different ratios of purified NK cells previously maintained overnight in RPMI 1640 complete medium supplemented with 50 U/ml of rhIL2 (*n* = 3). The results were expressed as means ± SEM. **(F,G)** Purified splenic NK cells were plated in complete medium supplemented with 5,000 U/ml rhIL2. **(F)** The living cell numbers and **(G)** the percentage of dead cells were determined daily by manual cell counting using trypan blue in Malassez chamber (*n* = 3). The results were expressed as mean ± SEM **(F)** and median ± Min to Max **(G)**. ns = not significant ^*^p ≤ 0.05 and ^**^p ≤ 0.005 using two-tailed non-parametric and unpaired Wilcoxon-Mann-Whitney test.

We analyzed the ability of splenic NK cells from FcRn^−/−^ and WT naive mice to produce IFN-γ and degranulate, which are major functions of NK cells. For this, we measured *de novo* IFN-γ production by intracellular staining of NK cells and NK cell degranulation by surface mobilization of CD107a, after exogenous stimulation with PMA/ionomycine and cytokines (IL-2 or IL-12, IL-15 and IL-18). Overall, NK cells from FcRn^−/−^mice produced less IFN-γ (Figures 7A,C) and expressed less CD107a on their surface (Figures 7B,D) than those from WT mice in all conditions. To gain insight into NK function in FcRn^−/−^ mice, we assessed NK cell cytotoxicity against YAC-1 cells in basal condition as described by Mizutani et al. (23). Although NK cells from FcRn^−/−^ animals had a significant lower expression of CD107a compared to WT mice (Figure 7D), there was no significant difference in NK cytoxicity against YAC-1 cells (Figure 7E). This result suggests that NK cells from an FcRn^−/−^ deprived microenvironment displayed similar “basal” cytotoxic properties when they are unstimulated. Additionally, we found that NK cells from FcRn^−/−^ and WT naive animals proliferated identically (Figure 7F), but died more in the presence of IL-2 5,000 U/mL (Figure 7G). Altogether, our results indicate that NK activation by cytokines is impaired in FcRn^−/−^ naive mice.

## Discussion

Herein, we confirmed the crucial role of FcRn in the anti-tumor immune response in the B16F10 model of experimental lung metastasis in which we clearly show an increase in lung metastasis in an FcRn-depleted environment in mice. Analysis of the immune cells infiltrating the lungs after intravenous injection of B16F10 cells revealed a decreased proportion of cDCs and CD8 T lymphocytes with lack of FcRn. These results agree with the already assessed function of FcRn-mediated tumor protection driven by DC and CD8^+^ T-cell activities described by Baker et al. (12). However, we identified NK cells as a new and additional cellular component of the FcRn-dependent anti-tumor response.

NK cells are effector lymphoid cells belonging to the innate immune system that can recognize and kill microbial-infected cells and play an important role in the immune surveillance against tumors (30). In this study, mice lacking FcRn showed reduced intratumoral NK-cell infiltration, which may participate in amplified development of B16F10 lung lesions, because NK cells are required for B16F10 tumor rejection (16, 23). In FcRn^−/−^ mice, NK cells had an immature phenotype on the whole, as characterized by surface markers (26). The particular cells affected were DP and CD27^−^ NK subtypes, which are able to proliferate under inflammation and exhibit effector functions.

The distribution of the four maturation stages depends on the tissue (31). In our B16F10 experimental lung metastasis model, the distribution of the NK cell subtypes was modified in lungs of FcRn^−/−^ mice and in the spleen to a lesser extent. These results suggest that the phenotypic alteration of NK cells is more pronounced in tumor-associated tissues. The alteration may be due to impaired *in situ* maturation in the tumor lung microenvironment caused by tumor-related soluble factors (32, 33). To understand the origin of the NK cell immature phenotype, we evaluated the distribution of NK cell subtypes in FcRn^−/−^ naive mice and found that they also displayed an immature phenotype in lungs as well as spleens. Although NK sub-populations are distributed differently in the spleen and lungs of mice (28) because of distinct homing properties and tissue-specific maturation, their distribution in the different peripheral organs of FcRn^−/−^ mice is unusual. Because NK cells develop from lineage-restricted progenitors in bone marrow, we analyzed NK subpopulations in this compartment. The proportion of NK precursors was higher in FcRn^−/−^ than WT mice, which suggests partial blockade of NK-cell development at early steps. This phenomenon confirmed the previously described hindrance effect of tumor growth on NK-cell maturation in bone marrow (17, 34) that we also observed in our lung experimental metastasis model in WT mice.

Because altered NK-cell maturation would affect the cellular properties of NK cells, we analyzed the ability of NK cells to synthesize IFN-γ and mobilize CD107a, which reflects NK-cell degranulation/cytotoxic activity. NK cells help eliminate B16F10 tumor cells in the experimental model of lung metastasis (16) and effector molecules, such as perforin and IFN-γ play important roles in NK-mediated inhibition of metastasis and tumor growth (17, 35). Although, non-activated NK cells from FcRn^−/−^ animals had a significant lower expression of CD107a compared to WT mice, it was not correlated with a reduced cytotoxicity efficacy. Interestingly, NK cells from FcRn^−/−^ mice were less prone to degranulate and synthesize IFN-γ after chemical stimulation and (IL-2 or IL-12, IL-15 and IL-18) cytokine activation than NK cells from WT mice. These cytokines are involved in proliferation, differentiation/maturation of NK cells and enhance their effector functions (36, 37). Pre-activation of NK cells with IL-2 or IL-12, IL-15 and IL-18 results in the generation of NK cells efficient to target and kill tumor cells (24, 38). The lower expression of CD107a and IFN-γ synthesis (after IL-2 or IL-12, IL-15, and IL-18 activation) as well as the increased cell death (in the presence of IL-2) in FcRn^−/−^ NK cells, support an impaired response of NK cells to cytokines in an FcRn^−/−^ deprived microenvironment. This might be critical to limit the spreading of lung tumor lesions in the B16F10 lung metastasis model. Accordingly, a lower CD107a expression was found in splenic NK cells from FcRn^−/−^ animals (compared to WT mice) in the B16F10 model and which persisted after cytokine activation (Supplemental Figure S6).

Once activated, NK cells produce cytokines and chemokines that regulate both the innate and adaptive immune system (39, 40). Because NK cells express no FcRn, the immature phenotype of NK cells might arise via indirect mechanisms due to the absence of FcRn in other cells of the immune system, such as DCs (10). Previous studies described interactions between NK cells and DCs and found a bidirectional crosstalk leading to NK-cell priming by DCs, which in turn induces DC maturation (41, 42). Knowing that FcRn-positive DCs are important for shaping the CD8^+^ effector T-cell anti-tumor response (12), FcRn might also affect the bidirectional cross-talk between NK cells and DCs. In light of our results showing impaired secretion of IFN-γ by NK cells in FcRn^−/−^ mice and the important role of IFN-γ in DC maturation by NK cells (43), IFN-γ might play a role in an FcRn-dependent cross-talk between NK cells and DC. Following this idea, the defective interleukin-12 level described in FcRn^−/−^ DCs (12) may also participate in the impaired NK-cell activation in our model because of its involvement in IFN-γ production by NK cells (41). Other FcRn-expressing cells, such as neutrophils and monocytes/macrophages (10, 44) may also be involved in NK-cell interactions (40, 45). Of note, we found a marked increase in the proportion of neutrophils in FcRn^−/−^ mice as compared with WT mice. Similarly, the proportion of neutrophils was enhanced in the model of anti-TNF antibody immunization in FcRn-deficient mice (46) and in other models (47). Previous studies also reported a high number of intratumoral neutrophils associated with the induction and maintenance of tumor angiogenesis (48, 49). Moreover, in breast cancer (50) and diverse murine cancer models (51, 52), macrophage/monocyte cells (53, 54) and/or neutrophils (52, 55) have been implicated in tumor promotion. From this evidence and our results, we cannot rule out a link between neutrophils expressing FcRn (44) and NK-cell maturation/activation. Whether this potential interaction occurred via soluble factors, such as cytokines or direct cell contact needs to be investigated. Finally, several cell types present in the hematopoietic niche or secondary lymphoid organs express FcRn. This is the case with stromal cells, endothelial and epithelial cells (44), which may also intervene in NK-cell maturation (36, 40, 56, 57). Further studies are needed to decipher the impact of FcRn-positive cells in the maturation of NK precursors.

For the first time, we described that NK-cell mediated tumor elimination/surveillance is impaired in an FcRn-deficient microenvironment. This could be linked to an abnormal cytokine response which needs further investigations to provide understanding in the involvement of FcRn. In light of our previous results (13) and those from Baker et al. (12), showing FcRn dysregulation in lung and colorectal cancers and associated with an unfavorable outcome, our current findings further support the central role of FcRn in anti-tumor immunity and highlight the interest of targeting FcRn for therapeutic purposes.

## Author contributions

DC performed the research, analyzed, discussed, interpreted data and literature and wrote the manuscript; CD, ED, LL, AV, CA, M-VD, DF, and CP contributed to the research and analyzed data; TB, NH-V, and VG-G supervised, discussed and verified data analysis and contributed to manuscript preparation.

### Conflict of interest statement

The authors declare that the research was conducted in the absence of any commercial or financial relationships that could be construed as a potential conflict of interest.
