# Supplementary material for: Lack of FcRn Impairs Natural Killer Cell Development and Functions in the Tumor Microenvironment
Source: Front Immunol. 2018 Sep 28;9:2259. doi: 10.3389/fimmu.2018.02259 (PMC6172308; doi:10.3389/fimmu.2018.02259)
Supplement: Supplementary file 1 [file Presentation_1.PPTX]

## Slide 1
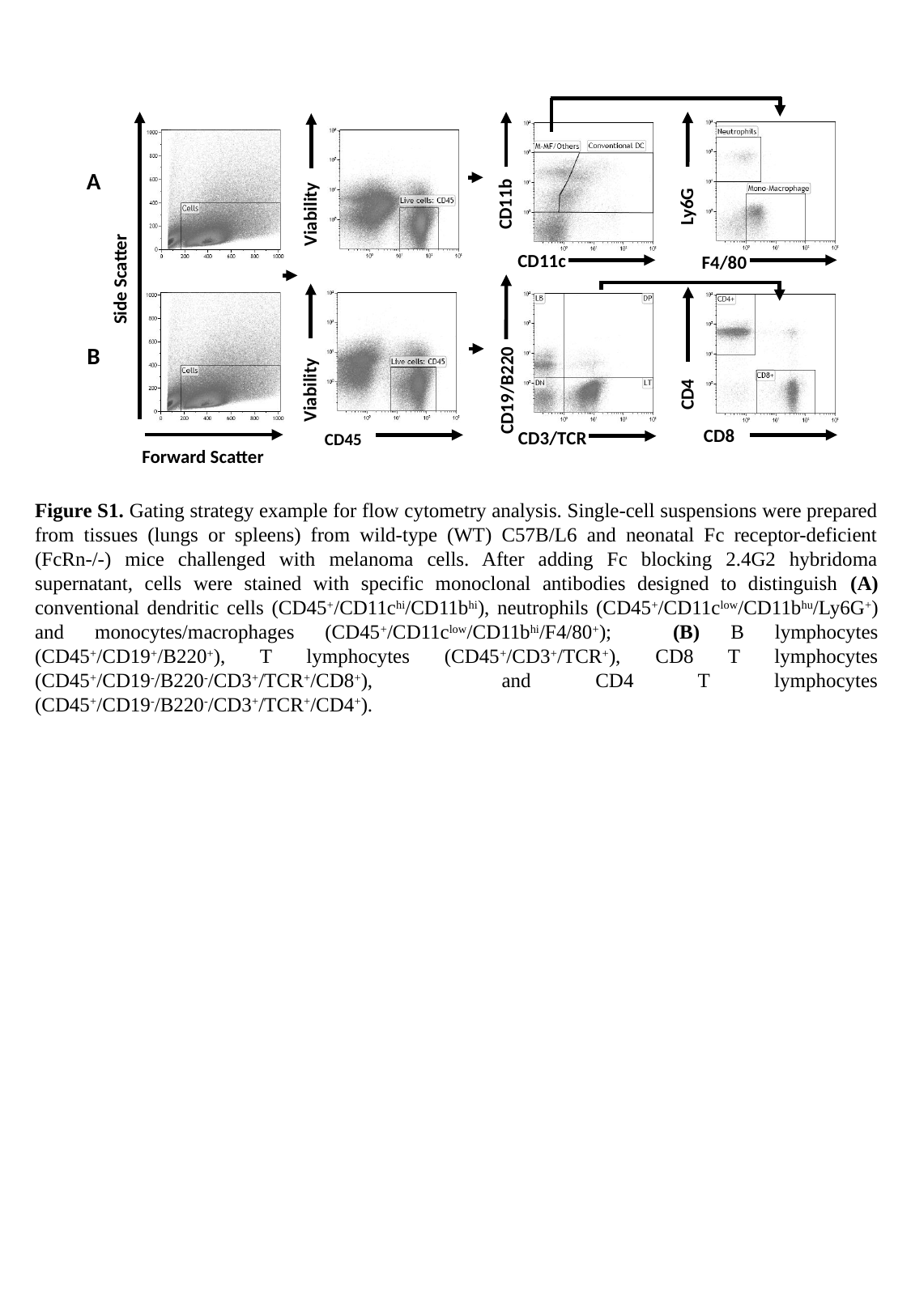

Side Scatter
CD11b
Ly6G
A
Viability
CD11c
F4/80
CD19/B220
CD4
B
Viability
CD8
CD3/TCR
CD45
Forward Scatter
Figure S1. Gating strategy example for flow cytometry analysis. Single-cell suspensions were prepared from tissues (lungs or spleens) from wild-type (WT) C57B/L6 and neonatal Fc receptor-deficient (FcRn-/-) mice challenged with melanoma cells. After adding Fc blocking 2.4G2 hybridoma supernatant, cells were stained with specific monoclonal antibodies designed to distinguish (A) conventional dendritic cells (CD45+/CD11chi/CD11bhi), neutrophils (CD45+/CD11clow/CD11bhu/Ly6G+) and monocytes/macrophages (CD45+/CD11clow/CD11bhi/F4/80+); (B) B lymphocytes (CD45+/CD19+/B220+), T lymphocytes (CD45+/CD3+/TCR+), CD8 T lymphocytes (CD45+/CD19-/B220-/CD3+/TCR+/CD8+), and CD4 T lymphocytes (CD45+/CD19-/B220-/CD3+/TCR+/CD4+).

## Slide 2
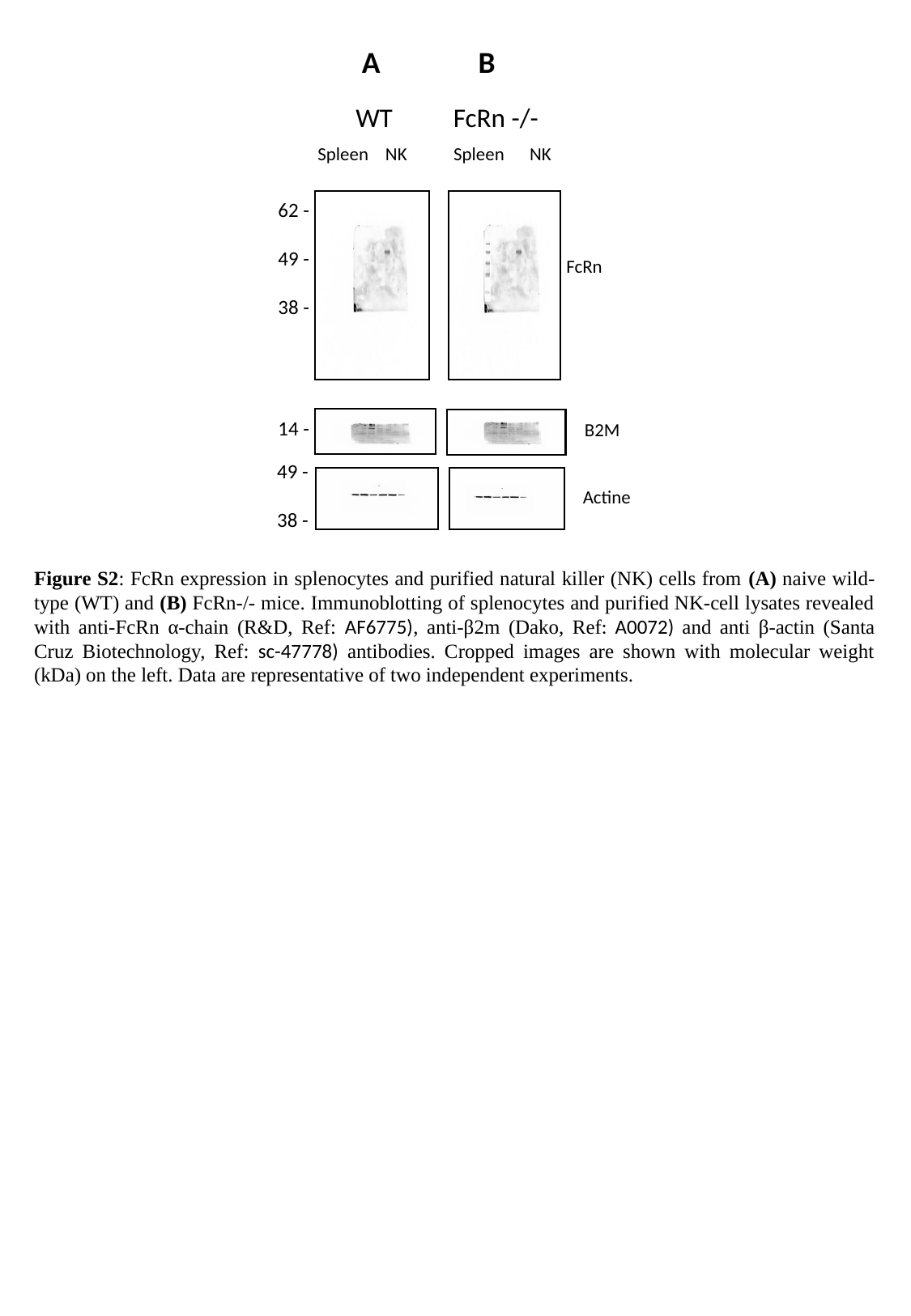

B
A
WT FcRn -/-
 Spleen NK Spleen NK
62 -
49 -
38 -
14 -
FcRn
49 -
38 -
B2M
Actine
Figure S2: FcRn expression in splenocytes and purified natural killer (NK) cells from (A) naive wild-type (WT) and (B) FcRn-/- mice. Immunoblotting of splenocytes and purified NK-cell lysates revealed with anti-FcRn α-chain (R&D, Ref: AF6775), anti-β2m (Dako, Ref: A0072) and anti β-actin (Santa Cruz Biotechnology, Ref: sc-47778) antibodies. Cropped images are shown with molecular weight (kDa) on the left. Data are representative of two independent experiments.

## Slide 3
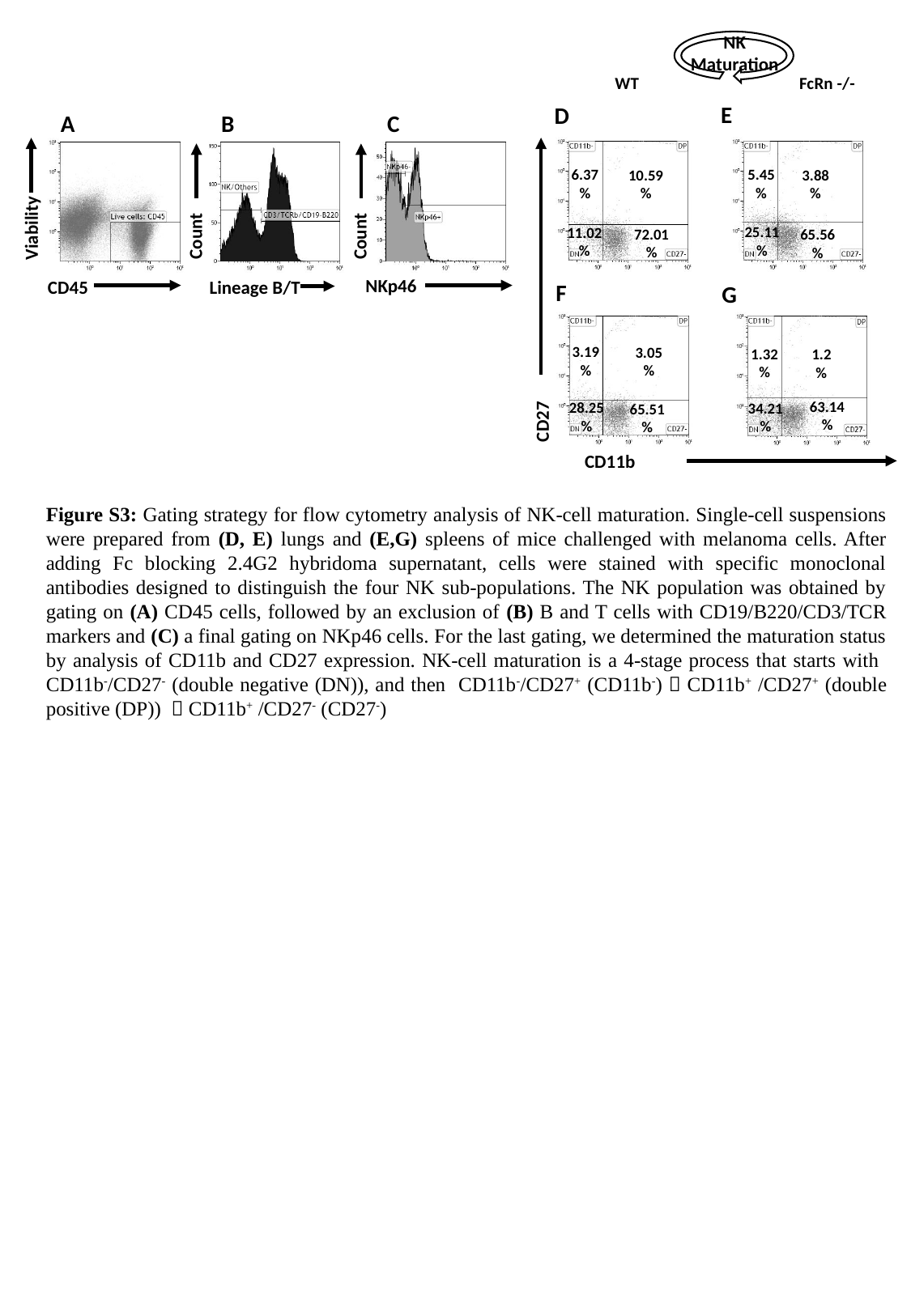

NK
Maturation
WT
FcRn -/-
E
D
A
B
C
Viability
CD27
Count
Count
6.37 %
5.45%
10.59 %
3.88
%
25.11%
11.02%
72.01%
65.56%
NKp46
CD45
Lineage B/T
F
G
3.19%
3.05 %
1.32%
1.2%
63.14%
28.25%
34.21%
65.51%
CD11b
Figure S3: Gating strategy for flow cytometry analysis of NK-cell maturation. Single-cell suspensions were prepared from (D, E) lungs and (E,G) spleens of mice challenged with melanoma cells. After adding Fc blocking 2.4G2 hybridoma supernatant, cells were stained with specific monoclonal antibodies designed to distinguish the four NK sub-populations. The NK population was obtained by gating on (A) CD45 cells, followed by an exclusion of (B) B and T cells with CD19/B220/CD3/TCR markers and (C) a final gating on NKp46 cells. For the last gating, we determined the maturation status by analysis of CD11b and CD27 expression. NK-cell maturation is a 4-stage process that starts with CD11b-/CD27- (double negative (DN)), and then CD11b-/CD27+ (CD11b-)  CD11b+ /CD27+ (double positive (DP))  CD11b+ /CD27- (CD27-)

## Slide 4
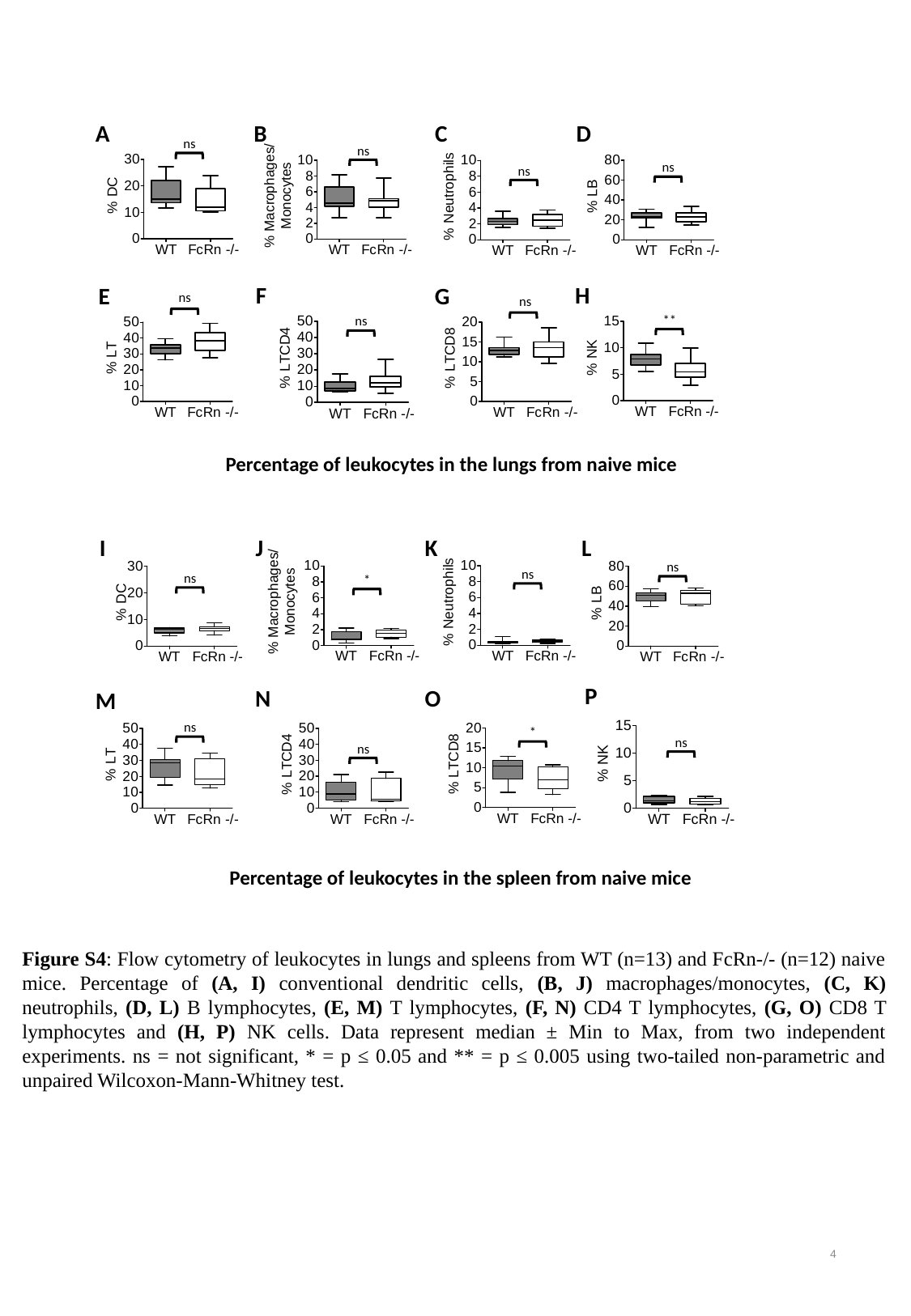

D
ns
C
ns
B
ns
A
ns
F
ns
H
**
E
ns
G
ns
Percentage of leukocytes in the lungs from naive mice
J
*
I
ns
L
ns
K
ns
P
ns
O
*
N
ns
M
ns
Percentage of leukocytes in the spleen from naive mice
Figure S4: Flow cytometry of leukocytes in lungs and spleens from WT (n=13) and FcRn-/- (n=12) naive mice. Percentage of (A, I) conventional dendritic cells, (B, J) macrophages/monocytes, (C, K) neutrophils, (D, L) B lymphocytes, (E, M) T lymphocytes, (F, N) CD4 T lymphocytes, (G, O) CD8 T lymphocytes and (H, P) NK cells. Data represent median ± Min to Max, from two independent experiments. ns = not significant, * = p ≤ 0.05 and ** = p ≤ 0.005 using two-tailed non-parametric and unpaired Wilcoxon-Mann-Whitney test.
4

## Slide 5
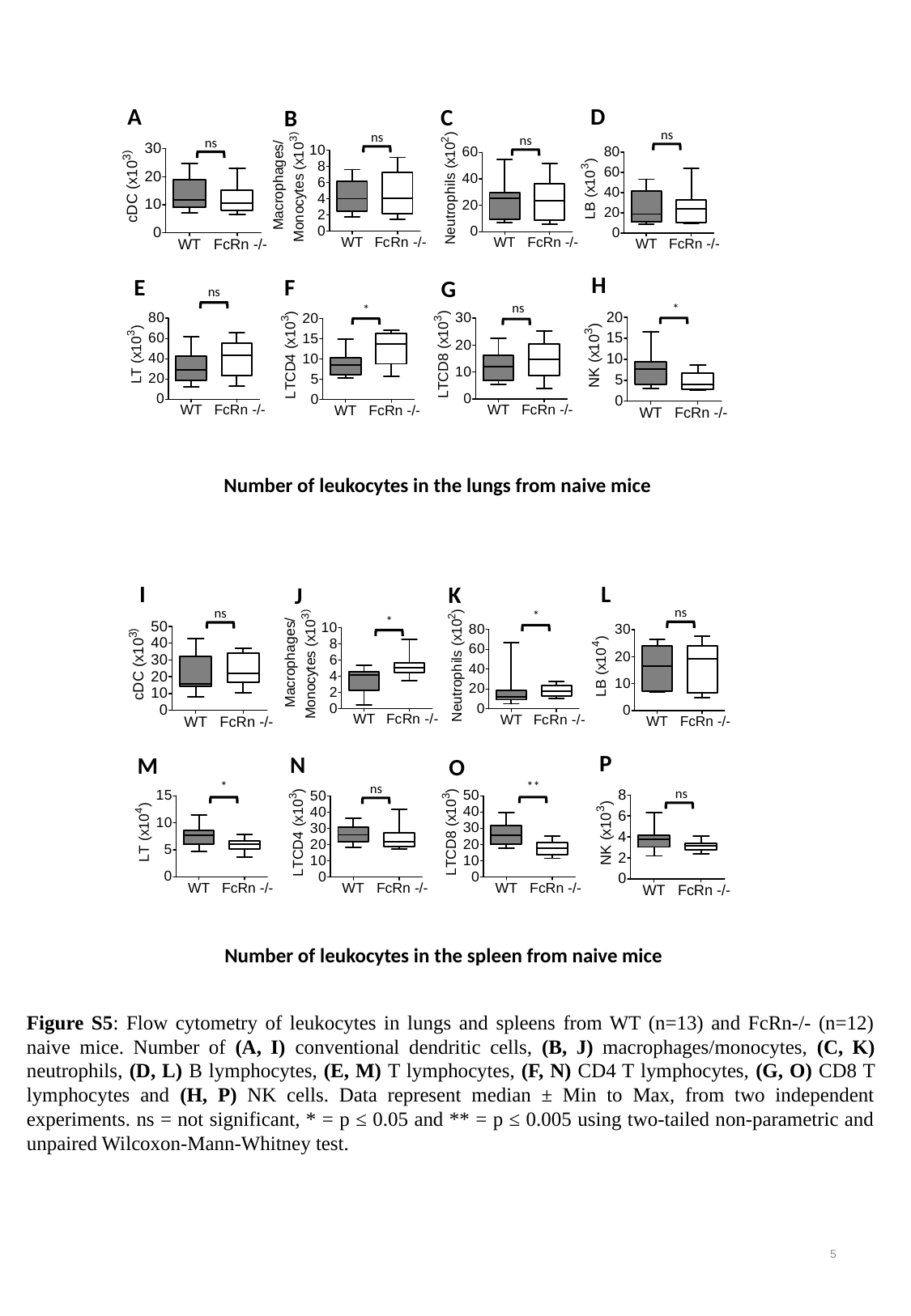

A
ns
D
ns
C
ns
B
ns
H
*
F
*
E
ns
G
ns
Number of leukocytes in the lungs from naive mice
I
ns
L
ns
K
*
J
*
P
ns
N
ns
M
*
O
**
Number of leukocytes in the spleen from naive mice
Figure S5: Flow cytometry of leukocytes in lungs and spleens from WT (n=13) and FcRn-/- (n=12) naive mice. Number of (A, I) conventional dendritic cells, (B, J) macrophages/monocytes, (C, K) neutrophils, (D, L) B lymphocytes, (E, M) T lymphocytes, (F, N) CD4 T lymphocytes, (G, O) CD8 T lymphocytes and (H, P) NK cells. Data represent median ± Min to Max, from two independent experiments. ns = not significant, * = p ≤ 0.05 and ** = p ≤ 0.005 using two-tailed non-parametric and unpaired Wilcoxon-Mann-Whitney test.
5

## Slide 6
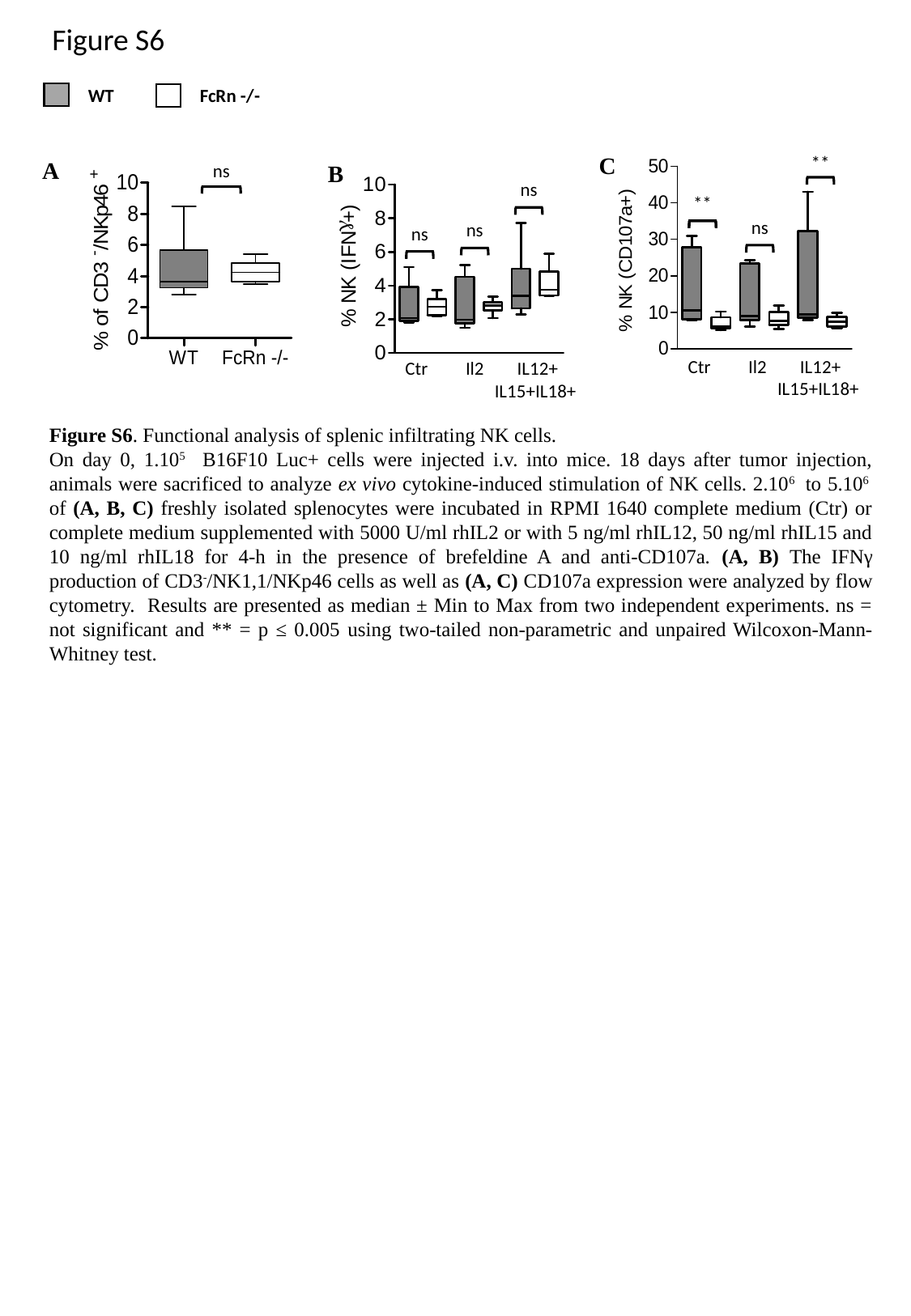

Figure S6
WT
FcRn -/-
C
**
**
ns
Ctr
Il2
IL12+
IL15+IL18+
A
ns
B
ns
ns
ns
Ctr
Il2
IL12+
IL15+IL18+
Figure S6. Functional analysis of splenic infiltrating NK cells.
On day 0, 1.105 B16F10 Luc+ cells were injected i.v. into mice. 18 days after tumor injection, animals were sacrificed to analyze ex vivo cytokine-induced stimulation of NK cells. 2.106 to 5.106 of (A, B, C) freshly isolated splenocytes were incubated in RPMI 1640 complete medium (Ctr) or complete medium supplemented with 5000 U/ml rhIL2 or with 5 ng/ml rhIL12, 50 ng/ml rhIL15 and 10 ng/ml rhIL18 for 4-h in the presence of brefeldine A and anti-CD107a. (A, B) The IFNγ production of CD3-/NK1,1/NKp46 cells as well as (A, C) CD107a expression were analyzed by flow cytometry. Results are presented as median ± Min to Max from two independent experiments. ns = not significant and ** = p ≤ 0.005 using two-tailed non-parametric and unpaired Wilcoxon-Mann-Whitney test.
